# Repurposing GLP-1 Receptor Agonists: A Perspective on Epigenetic Strategies to Combat Fibrosis and Hepatocellular Carcinoma in the Aged Liver

**DOI:** 10.3390/cancers17162600

**Published:** 2025-08-08

**Authors:** Silvia Hanna, Jason Sethiadi, Qazi Ali, Saloni Sinha

**Affiliations:** Weill Cornell Medicine, New York, NY 10021, USA; sih4008@med.cornell.edu (S.H.); jas4035@med.cornell.edu (J.S.); qaa4001@med.cornell.edu (Q.A.)

**Keywords:** GLP-1 receptor agonists, hepatocellular carcinoma (HCC), liver fibrosis, epigenetic reprogramming, DNA methylation, histone modifications, hepatic stellate cells (HSCs), metabolic dysfunction-associated steatotic liver disease (MASLD), cellular senescence, Sirtuin 1 (SIRT1)

## Abstract

The liver’s susceptibility to age-related diseases, including hepatocellular carcinoma (HCC), is increasingly linked to progressive epigenetic alterations that disrupt gene regulation, promote fibrosis, and impair regeneration. While glucagon-like peptide-1 receptor agonists (GLP-1RAs) are well-established in the treatment of type 2 diabetes and obesity, emerging evidence suggests they may also exert protective effects on the liver through the modulation of epigenetic pathways. In this perspective, we explore the hypothesis that GLP-1RAs may help restore a healthier epigenetic state in the aging liver by influencing mechanisms such as DNA methylation, histone modification, and non-coding RNA activity. These effects could reduce chronic inflammation, hepatic stellate cell activation, and fibrotic remodeling, key steps in the path to HCC. Preclinical studies have shown GLP-1RAs can affect transcriptional regulation and fibrotic markers, and early clinical data support improvements in liver function and structure in patients with metabolic liver disease. We highlight the need for further research to clarify these mechanisms in aging populations and propose that GLP-1RAs hold potential as a novel therapeutic strategy to reduce liver cancer risk by targeting the epigenetic contributors to disease progression.

## Simple Summary

Liver cancer is becoming more common in older adults, and current treatments are often administered too late or are too risky for this age group. This perspective explores how a class of medications called GLP-1 receptor agonists, commonly used to treat diabetes and obesity, may also help prevent liver cancer by targeting the root causes of liver damage. These drugs not only improve metabolism but may also reverse harmful changes in how liver cells turn genes on and off, a process known as epigenetic regulation. By reducing liver scarring and inflammation, they could slow the development of liver cancer. This research identifies a potentially novel application of an approved therapy for aging liver protection and underscores the need for further investigation to establish its safety and efficacy. If successful, this approach could offer hope for safer and more effective ways to protect aging livers and pave the way for treating other age-related diseases using similar strategies.

## 1. Introduction

**Figure 1:**
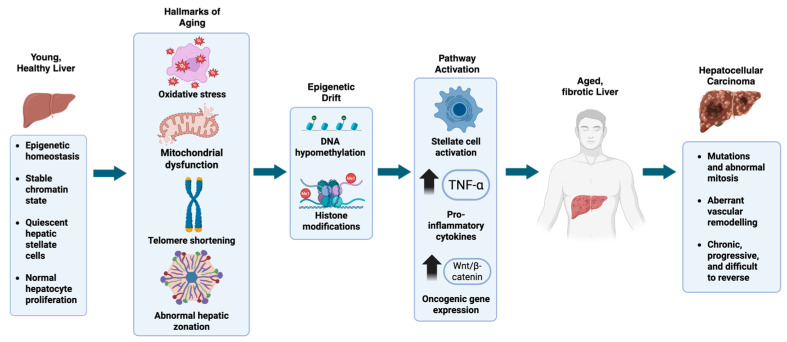
**Aging disrupts hepatic homeostasis and drives progression to fibrosis and hepatocellular carcinoma through epigenetic and inflammatory mechanisms**. In a young liver, homeostasis is maintained through a stable chromatin landscape, intact mitochondrial function, quiescent HSCs, and controlled hepatocyte turnover. With aging, oxidative stress and mitochondrial dysfunction emerge, contributing to telomere attrition and activation of the senescence-associated secretory phenotype (SASP). During stress and aging, hepatocyte zonation is distorted and expands abnormally, potentially impairing liver function. These hallmarks drive epigenetic drift, including global DNA hypomethylation, altered histone methylation and acetylation, and dysregulation of non-coding RNAs. These epigenetic disruptions upregulate pro-fibrotic mediators such as TGF-β1 and miR-21, which activate HSCs and promote extracellular matrix deposition. Concurrently, chronic inflammation and oncogenic reprogramming, partly mediated by increased TNF-α, IL-6, and aberrant activation of the Wnt/β-catenin pathway, support fibrosis progression and malignant transformation. As fibrosis advances, genomic instability and epigenetic silencing of tumor suppressors such as p53 and PTEN contribute to the development of HCC, characterized by abnormal mitosis, aberrant vascular remodeling, and resistance to regression.

Hepatocellular carcinoma (HCC) presents a significant and growing burden in aging populations worldwide. In the United States, there has been a substantial increase in the incidence and mortality of primary liver cancer, predominantly HCC, over the past two decades [1]. Projections indicate that the number of HCC cases in older adults is expected to continue rising [2]. Aging leads to pathological alterations in liver zonation and function, thereby increasing susceptibility to and accelerating the development of liver conditions such as metabolic dysfunction-associated steatotic liver disease (MASLD) and HCC [3,4]. These changes include expanded hepatic zones, reduced regenerative capacity, increased oxidative stress, mitochondrial dysfunction, and chronic low-grade inflammation, all of which contribute to liver vulnerability in older individuals [5,6] (Figure 1). 

The diagnosis, prevention, and treatment of HCC in older adults often present unique challenges that can impact treatment outcomes. HCC is generally identified at a later stage in older individuals compared to younger patients, potentially due to less frequent screening or symptoms being attributed to other age-related conditions [7]. Moreover, comorbidities complicate treatment decisions and increase the risk of adverse events [8].

Epigenetic dysregulation is a key driver of age-related hepatic decline. With age, the precise DNA methylation patterns that define cell identity and regulate gene expression begin to "drift." These changes accumulate over time and are exacerbated by chronic inflammation, a hallmark of aging, leading to altered gene expression that can impair liver function, further promote inflammation, fibrosis, and contribute to the progression of liver pathology [9,10]. For example, a common feature of cancer is global hypomethylation of the genome, leading to genomic instability and activation of oncogenes [11,12].

Glucagon-like peptide-1 receptor agonists (GLP-1RAs) have demonstrated substantial therapeutic success, initially revolutionizing the management of type 2 diabetes mellitus through their potent effects on glycemic control [13]. More recently, their profound efficacy in promoting significant body weight reduction has extended their application to the treatment of obesity [14]. Beyond these primary indications, GLP-1RAs have consistently shown beneficial effects on cardiovascular and renal outcomes, positioning them as cornerstone therapies for cardiometabolic diseases [15]. While their established benefits are clear, the precise molecular mechanisms underlying their broad protective effects, particularly in complex organs such as the liver, are still being fully elucidated. This warrants a deeper insight into the potential utility of GLP-1RAs in the epigenetic reprogramming of the aging liver.

## 2. Epigenetic Dysregulation in the Aging Liver

The aging liver exhibits a general trend of site-specific hypomethylation, particularly in certain repetitive elements and intergenic regions, which play a crucial role in maintaining genome integrity [16,17]. For example, in young, healthy cells, transposable elements are typically heavily methylated and epigenetically silenced to prevent their movement and potential disruption of gene function. However, this methylation pattern is disrupted in aged hepatocytes. These aging-associated epigenetic changes can lead to genomic instability [18]. This is similar to age-induced histone hypoacetylation, which is another key epigenetic alteration that impairs the plasticity of hepatocytes in the liver, diminishing their regenerative capacity [19].

Aging in the liver is accompanied by a gradual, gene-specific increase in DNA methylation in multiple tumor suppressor gene promoters, promoting hepatocarcinogenesis [20]. Numerous other studies have also demonstrated altered expression profiles of miRNAs in the aging liver, leading to widespread deregulation of target genes and contributing to oncogenesis [21]. For example, profiles of aged rat livers demonstrate an upregulation of miR-29a, miR-29c, miR-195 and miR-497 and a downregulation of miR-301a, miR-148b-3p, miR-7a, miR-93, miR-106b, miR-185, miR-450a, miR-539 and miR-301b. These miRNAs can target genes involved in cell cycle regulation, potentially contributing to the reduced proliferative capacity and increased senescence observed in aged hepatocytes [22]. In mice, the miR-465 family of X-linked miRNAs was found to be significantly upregulated with age, directly suppressing growth hormone receptor mRNA and downstream growth hormone signaling. This suppression lowers downstream IGF-1 production, which is involved in cell proliferation, tissue repair, and regeneration [23]. Therefore, the aging-associated changes in miRNA expression may impair the liver’s regenerative capacity. miRNAs also play roles in regulating inflammatory pathways and the activation of hepatic stellate cells. These cells are key contributors to liver fibrosis; when activated, they transform into myofibroblast-like cells that secrete collagen and other extracellular matrix proteins. In these cells, miR-21, miR-199a/b, and miR-221/222 were found to be upregulated in fibrosis, amplifying profibrotic TGF-β and PDGF signaling and stoking SMAD and collagen synthesis, thereby modulating key profibrogenic pathways [24]. Hence, in the aging liver, dysregulated miRNAs may contribute to the persistent activation of stellate cells, promoting their transition into myofibroblasts and exacerbating chronic inflammation and fibrotic progression.

A key outcome of these epigenetic shifts is the induction of cellular senescence [25]. While senescence initially prevents the uncontrolled proliferation of aged cells, it can lead to an oncogenic microenvironment when combined with chronic liver disease. Senescent hepatocytes can develop a pro-inflammatory secretome, known as the Senescence-Associated Secretory Phenotype (SASP). These senescent cells accumulate in the aged liver, creating a microenvironment that fosters inflammation, fibrosis, and the development of cancer [26,27].

## 3. Mechanisms of GLP-1 Receptor Agonists (GLP-1 RAs)

Glucagon-like peptide-1 (GLP-1) is an incretin hormone secreted in response to food intake that enhances insulin secretion, inhibits glucagon, delays gastric emptying, and promotes satiety. As native GLP-1 is rapidly degraded, synthetic GLP-1 receptor agonists (GLP-1RAs), including liraglutide and semaglutide, are used to treat type 2 diabetes and obesity [28].

Although canonical GLP-1R expression is not detected in the liver [29,30], GLP-1RAs benefit liver health and exert their influence on cancer risk indirectly through weight reduction, improved insulin sensitivity, and reduced hepatic lipid accumulation [31]. These changes may also lower systemic inflammation, particularly by reducing the release of inflammatory cytokines from adipose tissue [32,33,34].

GLP-1RAs may also act directly on inflammatory signaling in the liver by activating AMPK and suppressing NF-κB signaling, both of which intersect with transcriptional regulation [35]. This epigenetic influence has also been demonstrated in non-hepatic tissues. In cortical neurons, GLP-1R engagement activates the PI3K-AKT-CREB pathway to upregulate APE1 and accelerate base excision repair of oxidative DNA lesions [36]. At the same time, systemic GLP-1R agonism reverses age-related transcriptomic signatures across glial and neurovascular cell types [37]. In lung fibroblasts, GLP-1R agonists attenuate inflammation and fibrosis by disrupting NLRP3 inflammasome/PFKFB3-driven glycolysis, reducing lactate production, and suppressing histone lactylation at promoters of profibrotic genes such as Acta2 and Col1a1 thereby suppressing α-SMA and collagen I transcription [38]. If similar mechanisms of epigenetic modulation by GLP-1RAs are shown to extend to hepatic cell populations, they may contribute to long-term hepatic protection.

## 4. GLP-1RAs and Epigenetic Reprogramming in the Liver

GLP-1RAs are increasingly recognized for their profound positive influence on liver health, extending beyond their well-established roles in glucose regulation and weight management. While the beneficial effects on the liver are evident, the precise underlying mechanisms through which GLP-1RAs exert these hepatoprotective actions remain largely elusive. Current hypotheses suggest an indirect pathway, primarily mediated through the significant weight loss often observed with GLP-1RA therapy, which in turn can ameliorate various metabolic stressors on the liver [39]. However, this explanation may not fully encompass the breadth of their therapeutic impact.

A burgeoning area of research points to the critical involvement of epigenetic modifications in the etiology and progression of a spectrum of liver diseases, including MASLD, non-alcoholic steatohepatitis (NASH), cirrhosis, and HCC. These epigenetic alterations can significantly influence cellular processes within the liver. They are implicated in driving key pathological features, such as steatosis, inflammation, and fibrosis, ultimately leading to cancer development [40,41]. Understanding how these epigenetic landscapes are shaped and can be modulated is crucial for developing effective therapeutic strategies.

Recent research highlights the potential of targeting epigenetic mechanisms of GLP-1RAs like exenatide to treat liver disease through the modulation of histone deacetylases like SIRT1. SIRT1, a NAD^+^-dependent protein deacetylase, has been shown to exert protective effects against hepatic steatosis and inflammation, both key drivers in the progression from MASLD to more severe liver pathologies such as fibrosis and HCC. SIRT1 activation appears to restore metabolic homeostasis and reduce lipotoxic stress in the liver [42,43]. Therefore, the therapeutic benefits of GLP-1RAs may extend beyond glycemic control to include epigenetic reprogramming of hepatic tissue.

Some studies have also shown that GLP-1RAs may have a direct role in reshaping the epigenetic landscape in various other cell types. For example, treatment with exenatide in mice has been associated with the reversal of aging-related changes in gene expression in glial and neurovascular cells. These effects appear to span the entire genome, suggesting that GLP-1RAs may influence gene regulation on a broad, system-wide level [37]. While this observation was made in the brain, it provides a compelling precedent for the possibility that GLP-1RAs might exert similar genome-wide epigenetic effects in other organs, including the liver. 

While most benefits of GLP-1R expression in the liver may be indirect (e.g., through pancreatic insulin secretion affecting the liver), GLP-1RAs may also act on different liver cell types via receptor-independent mechanisms. For example, the degradation fragments of GLP-1, such as GLP-128–36 and GLP-132–36, may trigger a receptor-independent activation of the Wnt/β-catenin signaling pathway [44]. While the specific mechanism is not fully elucidated, it is hypothesized that these short, amphipathic peptides may cross the hepatocyte plasma membrane passively, or alternatively via an unidentified receptor or transporter [45]. Afterwards, they may utilize soluble adenylyl cyclase to activate PKA, leading to transcriptional reprogramming that suppresses gluconeogenesis and modulates lipid metabolism in the liver. Structurally, it is possible that liraglutide may also be cleaved to generate fragments that differ from GLP-128–36 and GLP-132–36 by only one amino acid residue [44]. These proposed mechanisms may explain how exenatide and liraglutide were found to reduce intrahepatic lipid in obese patients with hepatic stenosis in a manner not significantly correlated with reduction in total body weight, even in the absence of classical GLP-1R expression in hepatocytes [46]. Moreover, in a study combining semaglutide intervention with ex vivo exosome analyses, it was found that in patients who showed improvement in MASLD, isolated exosomes significantly modulated LX-2 stellate cell behavior, restoring viability and downregulating fibrogenic markers (α-SMA, p-SMAD2, CTGF, COL1A1, FN1). These findings suggest a receptor-independent mechanism whereby semaglutide alters systemic exosomal cargo to impact hepatic stellate cell activity [47]. 

Therefore, it is crucial to investigate epigenetic changes in specific liver cell types upon GLP-1RA exposure, even if the receptor expression is low or absent in some. Such modulation may help explain their beneficial effects on liver health, potentially independent of, or in addition to, their weight loss-inducing properties. Additionally, further research is warranted to elucidate the mechanisms by which GLP-1RAs enter hepatocytes, modulate epigenetics in liver disease, and restore healthier profiles that mitigate damage and promote regeneration. The specific effects of individual GLP-1RAs such as liraglutide, semaglutide, and tirzepatide on liver fibrosis, epigenetic remodeling, and HCC prevention are summarized in Table 1.

### Long Non-Coding RNAs Dysregulated in Liver Disease and Their Modulation by GLP-1RAs

Long non-coding RNAs (lncRNAs) have emerged as critical epigenetic regulators involved in liver aging, fibrosis, and hepatocellular carcinoma (HCC). Several lncRNAs exhibit aberrant expression patterns in diseased liver tissues, influencing key pathogenic pathways. For instance, HULC (Highly Upregulated in Liver Cancer) promotes hepatocarcinogenesis by sponging miR-372, thereby activating lipogenic and proliferative signaling cascades [50]. MALAT1 (Metastasis-Associated Lung Adenocarcinoma Transcript 1) is consistently overexpressed in liver fibrosis and HCC, functioning through recruitment of the histone methyltransferase EZH2 to silence tumor suppressor genes via H3K27 trimethylation [51,52]. In contrast, MEG3 (Maternally Expressed Gene 3) is downregulated in fibrotic and aging livers, where its loss correlates with diminished p53 activity and enhanced fibrogenic signaling [53]. These examples highlight the diverse regulatory roles of lncRNAs in liver pathology.

While direct evidence for GLP-1RAs in modulating hepatic lncRNAs is currently lacking, studies in other tissues suggest these drugs may influence lncRNA expression linked to fibrosis and inflammation. However, the specific effects of GLP-1RAs on liver lncRNAs remain poorly understood. Given the important regulatory roles of lncRNAs in liver aging and fibrosis, further focused research is needed to determine whether GLP-1RAs can modulate hepatic lncRNAs and whether this contributes to their therapeutic effects.

## 5. GLP-1RAs in Fibrosis and HCC Prevention

Liver fibrosis, a consequence of chronic liver injury, is characterized by the excessive accumulation of extracellular matrix (ECM) proteins, primarily collagen, leading to the formation of fibrous scars. This process is highly dynamic and can progress to cirrhosis and subsequently, HCC if the underlying cause persists [54]. As mentioned previously, a key event in hepatic fibrogenesis is the activation of HSCs under homeostasis. However, in response to liver injury and the resulting inflammatory signals, HSCs become activated and undergo transdifferentiation. During this transformation, they lose their quiescent, vitamin A-storing phenotype and acquire characteristics of myofibroblast-like cells. These activated HSCs begin to multiply, migrate to sites of injury, and develop contractile properties [55].

Activated HSCs become the primary producers of ECM components, including type I collagen (COL1A1) and tissue inhibitors of matrix metalloproteinases (TIMPs), which prevent the degradation of deposited collagen. A key pro-fibrogenic cytokine orchestrating this process is transforming growth factor-beta (TGF-β), particularly TGF-β1. TGF-β directly stimulates HSC activation and proliferation, promoting excessive secretion of ECM proteins [56].

Epigenetic mechanisms play a crucial role in controlling the expression of fibrogenic genes. For instance, specific histone modifications and DNA methylation patterns can influence the expression of genes like COL1A1 and TIMP1, thereby modulating the fibrotic response [57]. GLP-1RAs are becoming increasingly recognized for their pleiotropic effects, including significant anti-fibrotic properties. In various animal models of fibrosis, GLP-1RAs have been shown to attenuate fibrosis. For example, a long-acting dual agonist of GLP-1R and GLP-2R was found to improve hepatic fibrosis and may serve as a promising therapy to treat MASLD/NASH [49]. Additionally, these anti-fibrotic capabilities have been observed in organs other than the liver as well. Investigating pulmonary fibrosis models has shown that activating GLP-1R with liraglutide can attenuate inflammation and fibrosis [38].

**Figure 2:**
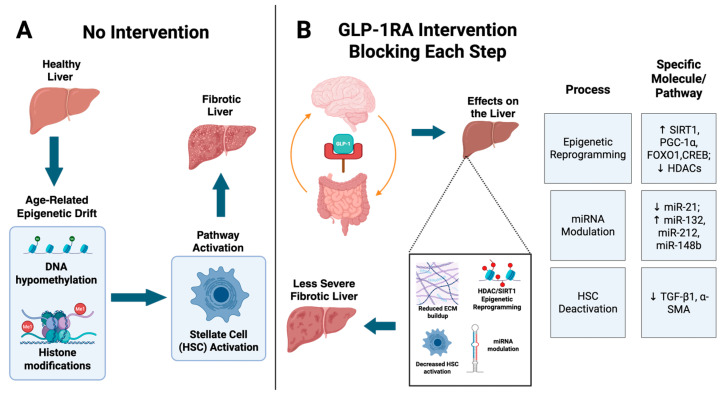
**GLP-1RAs block the progression from age-related epigenetic drift to liver fibrosis through multi-level molecular modulation**. Panel (**A**) shows the unopposed effects of biological aging on the liver, where epigenetic drift, including DNA hypomethylation and histone modifications, activates HSCs, promoting fibrogenesis and progression to a fibrotic liver. Panel (**B**) illustrates how GLP-1RA treatment, acting via the gut–brain–liver axis, disrupts this progression through epigenetic reprogramming (↑ SIRT1, PGC-1α, FOXO1, CREB; ↓ HDACs), microRNA modulation (↓ miR-21; ↑ miR-132, miR-212, miR-148b), and suppression of fibrotic signaling (↓ TGF-β1, α-SMA). These mechanisms collectively reduce HSC activation and ECM accumulation, resulting in a less fibrotic liver. The right-hand panel summarizes the targeted pathways and molecules involved in each process.

Clinically, these findings are supported by studies showing that liraglutide treatment induces histological resolution of NASH in 39% of patients versus 9% with placebo in patients with NASH [48]. Furthermore, early experimental data in diet-induced murine models of NASH suggest that the GLP-1RA IP118, alone or in combination with the FXR agonist obeticholic acid, significantly reduced liver mass, hepatic lipid accumulation, and circulating liver injury enzymes ALT and AST, reflecting attenuation of steatosis and hepatocellular damage [58]. While derived from non-human models and currently limited in external validity, the findings provide compelling preliminary insights that underscore the therapeutic potential of GLP-1-based therapies in the context of liver disease and warrant continued inquiry. Additionally, while GLP-1RAs have demonstrated epigenetic effects across various cell types, their specific mechanisms and capacity for epigenetic reprogramming in aged hepatic tissue remain incompletely characterized. Further research is needed to elucidate these pathways and confirm their relevance to age-associated hepatic epigenetic remodeling (Figure 2). 

## 6. Potential Clinical Implications and Future Directions

Older adults are particularly vulnerable to liver fibrosis and HCC due to age-related epigenetic changes and an exaggerated fibrogenic response to chronic liver injury. This makes them an ideal population for interventions targeting both fibrosis and cancer risk. With further research, GLP-1RAs, which are already FDA-approved for diabetes and obesity, may offer a promising therapeutic avenue given their emerging ability to modulate epigenetic pathways, such as inhibiting lactate-mediated histone lactylation in fibrotic contexts [38]. Early data suggest they may reduce HCC risk in patients with type 2 diabetes, pointing to potential synergy with existing HCC surveillance protocols [50].

Their known safety and tolerability profiles facilitate this specific style of clinical repurposing of GLP-1RAs [59,60]. Incorporating them into liver disease management will require precise patient stratification, using non-invasive fibrosis biomarkers (e.g., FIB-4, transient elastography) [61], and eventually, epigenetic signatures that predict responsiveness. This precision medicine approach could improve outcomes while minimizing unnecessary treatment. However, challenges remain, such as optimal dosing for liver-specific epigenetic modulation, which is not yet established, as current regimens target metabolic endpoints. Long-term safety in non-metabolic pathways, particularly in the context of aging and liver cancer prevention, needs further investigation. Additionally, genetic background, lifestyle factors, and disease etiology may influence individual responses, underscoring the need for predictive biomarkers.

To bridge the gap between preclinical promise and clinical application, future research should focus on mechanistic studies using aged liver models and patient biopsies to map epigenetic changes pre- and post-GLP-1RA treatment. This is important because it will clarify how GLP-1RAs exert their protective effects at a molecular level in aging livers, enabling targeted and effective interventions. Such insights are essential for designing clinical trials that identify the right patients, maximize therapeutic benefit, and minimize unnecessary treatment. Also, trials in older adults with advanced fibrosis should prioritize both histological and oncologic outcomes, while embedding biomarker discovery to guide personalized treatment strategies [62].

Ultimately, GLP-1RAs may offer benefits beyond liver disease, positioning them as potential anti-aging therapies. Their combined effects on metabolism, inflammation, and epigenetics make them attractive candidates for broader age-related disease prevention. Lastly, this is all coupled with the fact that the liver’s regenerative capacity and well-defined disease progression render it an ideal model for studying epigenetic rejuvenation.

## 7. Conclusions

The escalating global burden of HCC in aging populations underscores an urgent need for innovative preventive strategies. Based on preliminary research on their action in other tissues, GLP-1RAs may serve not merely as metabolic modulators, but also possibly as epigenetic reprogrammers capable of counteracting age-related dysregulation in the liver. We propose that these agents may mitigate the progression from chronic liver injury to fibrosis and ultimately to HCC by normalizing perturbed epigenetic landscapes in hepatic cells, thereby promoting a healthier cellular identity and function. However, further research is needed to establish the specific mechanisms of GLP-1RA epigenetic action in liver cells in the context of liver disease and to inform the development of standardized, optimized therapeutic protocols tailored to specific hepatic disorders. Additionally, such studies are warranted to confirm whether GLP-1RAs can produce clinically meaningful improvements in liver pathology, as direct experimental evidence of their efficacy in diseased hepatic tissue remains limited.

The clinical and public health potential of this paradigm is substantial. By leveraging the dual benefits of GLP-1RAs, which include their well-established metabolic improvements and their emerging role in epigenetic modulation, we have a unique opportunity to significantly reduce the HCC burden, particularly in vulnerable older adults. Their ability to ameliorate hepatic steatosis, inflammation, and fibrosis, combined with potential direct impacts on oncogenic pathways through epigenetic mechanisms, offers a multi-pronged approach to liver cancer prevention. This is particularly salient given the challenges of HCC management in older, often comorbid, patients.

Ultimately, this perspective suggests a crucial paradigm shift in our understanding and application of GLP-1RAs. Moving beyond their established role in glycemic control and weight management, we envision their strategic use as epigenetic therapies in geriatrics, specifically targeting age-related liver pathologies. This not only opens new avenues for HCC prevention but also highlights the liver as a compelling model for exploring epigenetic rejuvenation, potentially informing interventions for other age-related diseases. Future research will be crucial in unlocking the full potential of GLP-1RAs in promoting a healthier and more resilient aging liver.

## Figures and Tables

**Table 1:** Comparative effects of liraglutide, semaglutide, and tirzepatide on liver fibrosis, inflammation, and hepatocellular carcinoma (HCC) risk.

GLP-1RA	Mechanism of Action	Effects on Liver (Aged/HCC)	Model/Study Type	Reference
Liraglutide	GLP-1 receptor agonist (daily)	Histological resolution of NASH and metabolic improvement; potential epigenetic modulation via SIRT1	Diet-induced NASH models; human clinical trial	[48]
		Reduces pro-fibrotic histone lactylation	Pulmonary fibrosis model (lung fibroblasts)	[38]
Semaglutide	GLP-1 receptor agonist (weekly)	Exosomes suppress SMAD2 phosphorylation in HSCs; ↓ ECM gene expression and fibrosis markers–improving liver fibrosis in MASLD	Human ex vivo + LX-2 hepatic stellate cells	[47]
Tirzepatide	Long-acting dual agonist of GLP-1 and GLP-2 receptors	Stronger metabolic improvements; ↓ steatosis, inflammation, and hepatic fat accumulation	Preclinical NASH models; clinical trials in HCC	[49,50]

## Data Availability

The data presented are available in the references cited in this article.
